# Widespread Hyper-Coupling and Frequency-Specific Dysregulation of Phase-Amplitude Coupling in Young Children with Autism Spectrum Disorder

**DOI:** 10.3390/brainsci16070718

**Published:** 2026-07-04

**Authors:** Jiannan Kang, Zongbing Xiao, Zhiyuan Fan, Xiangyu Zhang, Xiaoli Li, Xing Jin

**Affiliations:** 1 College of Electronic & Information Engineering, Hebei University, Baoding 071002, China; kangjiannan81@163.com (J.K.); zongbingxiao@163.com (Z.X.); 13613598764@163.com (Z.F.); 13633195227@163.com (X.Z.); 2 State Key Laboratory of Cognitive Neuroscience and Learning, Beijing Normal University, Beijing 100875, China; xiaoli@bnu.edu.cn; 3 School of Artificial Intelligence, North China University of Science and Technology, Beijing 063210, China

**Keywords:** autism spectrum disorder, PAC, resting-state EEG, neural oscillations

## Abstract

**Highlights:**

**What are the main findings?**

- Children with ASD showed widespread, frequency-specific PAC abnormalities, especially increased δ/θ/α–γ coupling across local and long-range brain networks.
- β-band coupling showed a mixed pattern: δ–β coupling was increased in several regions, while α–β coupling was reduced mainly in frontal and central areas.

**What are the implications of the main findings?**

- PAC may serve as a useful EEG-based marker for atypical neural coordination in young children with ASD.
- The findings suggest ASD-related brain dysregulation is not uniform, but depends on specific frequency bands and brain regions, with some PAC measures linked to behavioral symptom severity.

**Abstract:**

Background: Autism spectrum disorder (ASD) is characterized by widespread aberrations in brain scalp-level synchronization. Phase-amplitude coupling (PAC), which reflects cross-frequency neuronal oscillatory interactions, serves as a crucial metric for assessing functional brain integration. However, the specific patterns of PAC at both intra-region and inter-region scalp levels in young children with ASD, as well as their precise associations with clinical symptoms, remain unclear. Methods: This study enrolled 237 children with ASD aged 3–9 years and 201 age-matched typically developing (TD) children. Resting-state electroencephalography (EEG) data were acquired from all participants. The analysis systematically examined low-frequency oscillation phase (δ, θ, α) modulation of high-frequency oscillation amplitude (β and low γ) from both intra-region and inter-region dimensions. The PAC strength was quantified using the modulation index (MI). Multiple comparisons were corrected using the Bonferroni method. Finally, correlations between PAC metrics and Autism Behavior Checklist (ABC) scores were analyzed. Results: Compared to the control group, children with ASD exhibited significant frequency-specific PAC abnormalities: (1) Multi-regional γ hyper-coupling: There was a significant enhancement in the modulation of γ amplitude by δ/θ/α phase across the measured scalp regions, suggesting abnormal high-frequency synchronization. (2) Dissociated β modulation patterns: The ASD group showed increased δ–β coupling (predominantly in frontal, temporal, and occipital lobes) alongside significantly reduced α–β coupling (localized to frontal and central regions). This reflects both an abnormal locking of slow-wave activity to the β band and a diminished regulatory role of α oscillations. (3) Clinical correlation: Notably, abnormally elevated PAC strength (particularly in the δ/θ/α–γ bands) showed a negative correlation with clinical symptom severity—that is, stronger coupling was associated with lower scores on the ABC. Conclusions: Leveraging a large-sample dataset, this study characterizes the landscape of aberrant cross-frequency interactions in young children with ASD. Our findings indicate that the neuroelectrical activity in ASD goes beyond mere connectivity anomalies by demonstrating altered PAC strength at both the intra-region and inter-region levels. Notably, the strength of this aberrant intra-region PAC is correlated with clinical symptoms.

## 1. Introduction

Autism Spectrum Disorder (ASD) is a pervasive neurodevelopmental condition characterized by persistent deficits in social communication and interaction, alongside restricted, repetitive patterns of behavior, interests, or activities [1,2]. The heterogeneous clinical presentation and etiological complexity of ASD highlight the ongoing need for objective neurophysiological markers that can contribute to early identification and a deeper understanding of its underlying neural atypicalities.

Electroencephalography (EEG) offers a non-invasive and high-temporal-resolution approach to investigate brain function, making it particularly suitable for pediatric populations [3]. While previous EEG studies in ASD have revealed alterations in spectral power and functional connectivity [4,5], these analyses often overlook the intricate cross-frequency interactions crucial for neural information processing. Phase-Amplitude Coupling (PAC), which quantifies the modulation of high-frequency oscillatory amplitude by the phase of lower-frequency oscillations, represents a fundamental mechanism for neural communication and functional integration within and between brain regions [6,7].

However, existing literature on PAC in ASD, particularly in young children, remains inconsistent. While some studies reported elevated PAC [8,9,10], others found significant reductions [11,12] or complex, region-specific patterns [13] involving different frequency pairs. Furthermore, many existing findings are limited by small sample sizes or the technical challenges associated with processing noisy pediatric EEG data, and the utility of low-density EEG systems in capturing robust PAC features relevant to ASD clinical phenotypes warrants further investigation. To address these gaps, this study employed an 8-channel resting-state EEG system to meticulously analyze PAC patterns. We recruited a large cohort of 237 young children with ASD and 201 typically developing (TD) controls. The primary aims were to identify widespread and frequency-specific alterations in intra-region PAC in young children with ASD and to explore the linear relationship between these electrophysiological abnormalities and total scores on the Autism Behavior Checklist (ABC).

## 2. Materials and Methods

### 2.1. Participants

A total of 438 children were recruited for this study and divided into an ASD group and a TD control group. The ASD group comprised 237 children (183 males, 54 females; age range: 3–9 years, mean age: 5.33 ± 1.41 years), and the TD group comprised 201 children (154 males, 47 females; age range: 3–9 years, mean age: 5.26 ± 1.23 years). Although the total sample sizes differed between groups (ASD: *n* = 237; TD: *n* = 201), chi-square tests revealed no significant bias in the distributions of age (χ^2^(5) = 1.884, *p* = 0.865) or gender (χ^2^(1) = 0.022, *p* = 0.882). This ensured that the group comparisons were not confounded by age or gender. Detailed information is presented in Table 1. Participants in the ASD group were recruited from multiple rehabilitation and special education institutions across China. All children with ASD were formally diagnosed by professional psychiatrists according to the Diagnostic and Statistical Manual of Mental Disorders, Fifth Edition (DSM-5) criteria [14]. Children in the TD group were recruited from local kindergartens and elementary schools. They were screened to confirm the absence of a family history of psychiatric disorders, epilepsy, or traumatic brain injury. To ensure data validity and safety, stringent inclusion and exclusion criteria were applied. For the ASD group, the participants were required to: (1) meet the DSM-5 diagnostic criteria and have a confirmed clinical diagnosis; (2) have not taken antipsychotic or anticonvulsant medications immediately before or during the study, and have no prior history of brain stimulation therapy; (3) have no metallic implants (e.g., cochlear implants, metal stents); and (4) have no history of brain injury or other comorbid neurodevelopmental disorders. Prior to the commencement of the experiment, all study procedures were explained in detail to the participants and their legal guardians, who subsequently provided written informed consent. The study was conducted in accordance with the Declaration of Helsinki and was approved by the Ethics Committee of Ningbo Rehabilitation Hospital (Approval Number: 2023004). There were no statistically significant differences in age or gender between the typically developing children and the ASD group. The detailed specific demographic information of the participants is shown in Table 1.

### 2.2. EEG Data Acquisition

The resting-state EEG data were acquired using an 8-channel EEG acquisition system. Prior to the experiment, the participants (and their guardians) were instructed to ensure sufficient sleep the night before and to wash their scalps to optimize signal quality. Data collection took place in a sound-attenuated and quiet laboratory. Participants were seated comfortably in a chair and instructed to remain relaxed, awake, and at rest with their eyes open. Electrode placement followed the international 10–20 system, with a total of 8 scalp channels recorded: left/right frontal (F3, F4), left/right temporal (T3, T4), left/right central (C3, C4), and left/right occipital (O1, O2). Conductive gel was applied to optimize electrode contact. The Cz electrode served as the online reference, and a GND electrode was used as ground. The sampling rate was set at 1000 Hz, and the impedance at all electrodes was maintained below 20 kΩ throughout the recording. Under the supervision of a trained technician, a minimum of 5 min of continuous EEG data were recorded for each participant.

### 2.3. EEG Signal Preprocessing

The offline preprocessing of the raw data was performed using MATLAB R2024 and the EEGLAB v2022.0 toolbox, following a rigorous pipeline designed to optimize signal quality for subsequent PAC analysis. First, a 50 Hz notch filter was applied to eliminate powerline interference. Second, a 0.5–45 Hz bandpass filter was used to remove low-frequency drifts and high-frequency noise while preserving the primary EEG rhythms. Based on this upper cutoff frequency, the low γ band was strictly defined as 30–45 Hz. Third, to effectively mitigate physiological artifacts that could otherwise obscure genuine neural interactions, we employed the Ensemble Empirical Mode Decomposition–Independent Component Analysis (EEMD-ICA) algorithm [15]. This advanced method was chosen due to its demonstrated superiority in handling the non-linear and non-stationary characteristics often present in pediatric EEG signals, and its capacity for robust artifact rejection. Specifically, EEMD adaptively decomposes the raw EEG into a set of intrinsic mode functions (IMFs), which more accurately represent the signal’s instantaneous frequency and amplitude compared to traditional Fourier-based methods. This decomposition is crucial for preserving the integrity of phase and amplitude relationships, which are paramount for accurate PAC estimation. Subsequently, ICA is applied to these IMFs to meticulously identify and remove components attributed to common physiological artifacts, including ocular (blinks, horizontal eye movements), cardiac, and muscular sources. Specifically, because high-frequency EEG analysis is inherently susceptible to myogenic contamination, independent components exhibiting peripheral topographical distributions and characteristic broad-band high-frequency noise without distinct neural peaks were explicitly identified as electromyographic (EMG) artifacts and rigorously removed. By providing a cleaner signal with minimized artifactual contributions, EEMD-ICA enhances the reliability and interpretability of the subsequent PAC calculations, ensuring that observed couplings are more likely to reflect genuine neurophysiological processes. Finally, any remaining noise segments containing significant motion artifacts or bad channels were manually rejected. The data were then re-referenced, and the resulting clean dataset was used for all subsequent calculations and analyses.

### 2.4. Phase-Amplitude Coupling

The MI based on the KL divergence was employed to quantify the strength of PAC [7,16,17]. A comodulogram was used to characterize multi-frequency PAC in both the ASD and TD groups to identify the specific frequency bands of interest. The calculation of PAC proceeded as follows. First, the raw signal, defined as *X*(*t*), was bandpass-filtered using the *eegfilt.m* function from the EEGLAB toolbox to extract the narrowband low-frequency signal *X_L_*(*t*) and high-frequency signal *X_H_*(*t*). Subsequently, the Hilbert transform was applied to extract the instantaneous phase *φ_L_*(*t*) from *X_L_*(*t*) and the instantaneous amplitude *A_H_*(*t*) from *X_H_*(*t*). To analyze the dynamic changes in coupling, we segmented the continuous phase and amplitude sequences into k sliding time windows. We select a 80-s window length for the resting-state data to obtain stable and reliable PAC estimates, effectively avoiding false-positive results that stem from insufficient sampling of the phase-amplitude distribution [8]. Furthermore, we apply a high overlap rate of 90% to mitigate the loss of temporal specificity inherent in the longer window, allowing us to track coupling dynamics smoothly and continuously over time. Within each time window *k*, the phase range [−*π*,*π*] was divided evenly into *m* = 18 bins. The corresponding instantaneous amplitude *A_H_*(*t*) at each time point was assigned to its respective phase bin *j* based on the instantaneous phase value *φ_L_*(*t*). The average amplitude ${\overset{-}{A}}_{H_{k}}(j)$ within each bin *j* was then calculated. To construct the time-varying phase-amplitude distribution *M_k_*, the average amplitudes were normalized using the formula:

(1) $$M_{k}(j)\;=\;{\overset{-}{A}}_{H_{k}}(j)/\sum_{j=1}^{m}{\overset{-}{A}}_{H_{k}}(j)$$

The MI, based on the distance, was used to quantify PAC strength [7,17]. This metric measures the degree to which the observed phase-amplitude distribution *M_k_* deviates from a uniform distribution *U*. The KL distance is defined as:

(2) $$D_{\mathrm{KL}}(M_{k},\;U)\;=\;\sum_{j=1}^{m}M_{k}(j)\log[M_{k}(j)/U(j)]$$

where U(j) = 1/m represents the uniform distribution. The MI is then defined as:

(3) $$\mathrm{MI}\;={\;D}_{\mathrm{KL}}(M_{k},\;U)/\log(m)$$

The formula can be simplified to:

(4) $$\mathrm{MI}\;=\;\sum_{j=1}^{m}M_{k}(j)\log M_{k}(j)/\log(m)+1$$

The MI value ranges from 0 to 1, where 0 indicates no coupling (uniform distribution) and 1 represents maximal (or perfect) phase-amplitude locking [18]. Finally, the MI values across all corresponding time windows for each participant were averaged to obtain a single, representative MI value per subject for all subsequent experimental analyses. While formal surrogate-data procedures were computationally prohibitive for this large-scale dataset, our methodology incorporated inherent safeguards against spurious coupling arising from non-sinusoidal waveform asymmetries. First, the EEMD algorithm is specifically designed to handle non-linear and non-stationary signals, extracting intrinsic mode functions that more faithfully preserve true physiological oscillatory shapes without introducing severe filtering artifacts. Second, the KL divergence-based MI is demonstrably robust against transient amplitude outliers and shape asymmetries compared to other traditional metrics.

Unlike previous PAC analyses, we examined both ASD and TD groups for two distinct types of PAC: (1) the intra-region PAC within the same brain region (i.e., coupling between low-frequency phase and high-frequency amplitude from the same channel, reflecting local dynamics within a region), which we refer to as intra-region PAC; and (2) the inter-region PAC between different brain regions (i.e., PAC calculated from pairs of channels located in distinct regions), which we term inter-region PAC.

### 2.5. Statistical Analysis

All statistical analyses and data visualization were performed using MATLAB (MathWorks, Natick, MA, USA) and Origin 2024 (OriginLab, Northampton, MA, USA). For demographic characteristics, chi-square tests were used to analyze the distribution differences in age and gender between groups. For the comparison of EEG-derived metrics, differences in PAC MI values between groups were assessed using independent samples *t*-tests. Additionally, to control for Type I errors across multiple comparisons, for both intra-region and inter-region PAC analyses, we conducted Bonferroni correction. Specifically, the uncorrected *p*-values were multiplied by the total number of independent tests conducted in each analytical domain (e.g., *n* = 5 for the intra-region comparisons, and *n* = 20 for the inter-region comparisons). For homologous bilateral pairs (i.e., F3/F4, C3/C4, and O1/O2), the two corresponding electrodes were pooled together to form a composite region. Results were considered statistically significant only if the adjusted *p*-value remained below the 0.05 threshold. To quantify the practical effect sizes of the group differences and facilitate the interpretation of their biological relevance, Cohen’s d values were calculated. The effect size thresholds were defined as follows: |d| ≥ 0.2 for a small effect, |d| ≥ 0.5 for a medium effect, and |d| ≥ 0.8 for a large effect. Furthermore, Pearson’s product-moment correlation analysis was conducted to quantify the linear relationship between the PAC MI values and the total scores on the ABC. To stringently control for Type I error, these analyses were strictly restricted exclusively to the 21 specific region-frequency pairs that had demonstrated significant between group differences. The statistical significance of the correlation coefficients (*H*_0_: *ρ* = 0) was determined using a two-tailed Student’s t-distribution. All resulting uncorrected *p*-values were subjected to Bonferroni correction across the 21 independent tests (*n* = 21), with statistical significance defined as an adjusted *p* < 0.05. In all figures, statistical significance levels of *p* < 0.05, *p* < 0.01, and *p* < 0.001 are annotated with the symbols “*”, “**”, and “***”, respectively.

### 2.6. AI Usage Disclosure

AI-assisted tools Gemini-Nano-Banana-2 were used only to assist with the layout, formatting, and aesthetic arrangement of the graphical abstract. The underlying scientific concepts, data, interpretation, and original sketches were developed and provided by the authors. No AI tools were used for study design, data collection, data analysis, data interpretation, or the generation of scientific conclusions.

## 3. Results

### 3.1. Identification of Key Frequency Bands Based on Group-Averaged PAC Comodulograms

**Figure 1:**
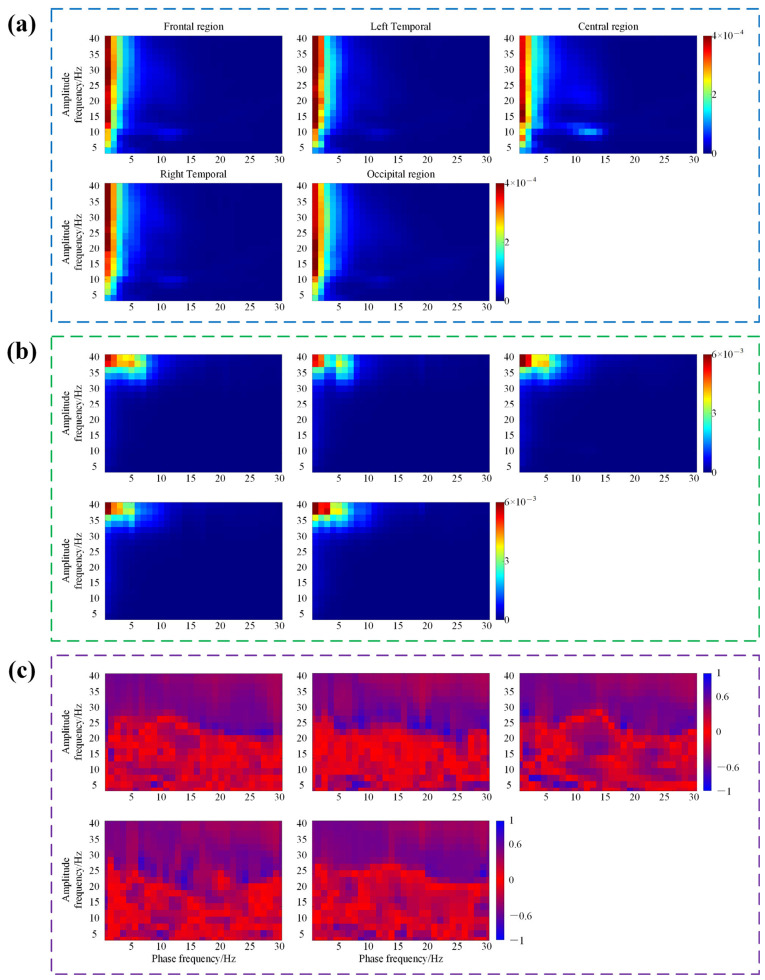
Group-averaged PAC comodulograms and effect size maps across all brain regions. Panels (**a**) and (**b**) display the average PAC strength MI for the TD and ASD groups, respectively, across five representative brain regions (frontal lobe, left temporal lobe, central region, right temporal lobe, occipital lobe). The x-axis represents the low-frequency phase (*f_P_*), and the y-axis represents the high-frequency amplitude (*f_A_*). Warmer colors indicate stronger coupling. Panel (**c**) shows the corresponding effect size Cohen’s d heatmap for the between-group differences. Larger absolute values of Cohen’s d indicate more pronounced group differences.

Figure 1 presents the group-averaged PAC comodulograms and the corresponding effect size maps for the five regions of interest: the frontal lobe, left temporal lobe, central region, right temporal lobe, and occipital lobe. The comodulograms for both the TD group (Figure 1a) and the ASD group (Figure 1b) consistently revealed cross-frequency interactions across all examined brain regions. Specifically, the phase of low-frequency bands (δ, θ, α) modulated the amplitude of high-frequency bands (β, γ), as indicated by the phase-frequency (x-axis) and amplitude-frequency (y-axis) dimensions in Figure 1a,b. A visual comparison between panels (a) and (b) suggests that the group differences were most pronounced for the modulation of the γ-band amplitude by the δ, θ, and α band phases. To identify the specific frequency pairs underlying these observed group differences, an effect size map was generated (Figure 1c). Visual inspection indicated that the most substantial differences between the ASD and TD groups were localized to frequency combinations where the phase of δ, θ, or α bands modulated the amplitude of β or γ bands. Consequently, all subsequent statistical comparisons and region-specific analyses focused on the following six coupling pairs: δ–β, θ–β, α–β, δ–γ, θ–γ, and α–γ.

### 3.2. Frequency-Specific Alterations in Intra-Region PAC

**Figure 2:**
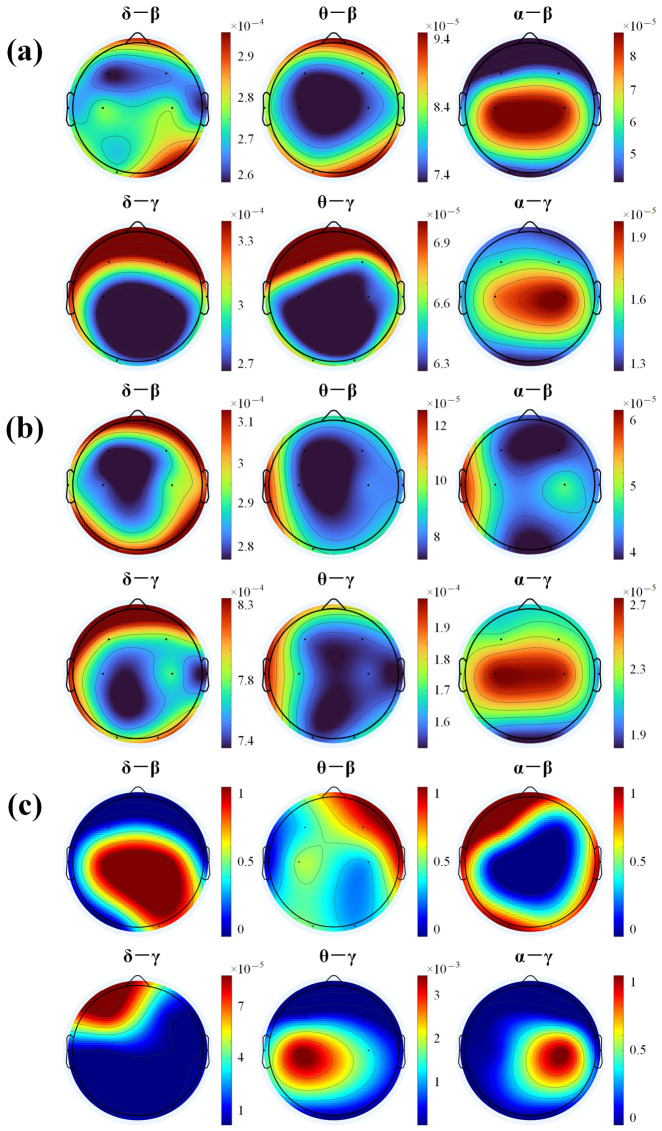
Topographic distribution and statistical comparison of intra-region PAC strength. Panels (**a**) and (**b**) show the average MI topographic maps for the TD and ASD groups, respectively. The top row in both panels displays the PAC distribution for the δ–β, θ–β, and α–β frequency pairs, while the bottom row displays the distribution for the δ–γ, θ–γ, and α–γ pairs. Panel (**c**) presents the spatial distribution of Bonferroni corrected *p*-values from independent-samples *t*-tests between the two groups, with the arrangement of frequency pairs consistent with the maps above.

**Figure 3:**
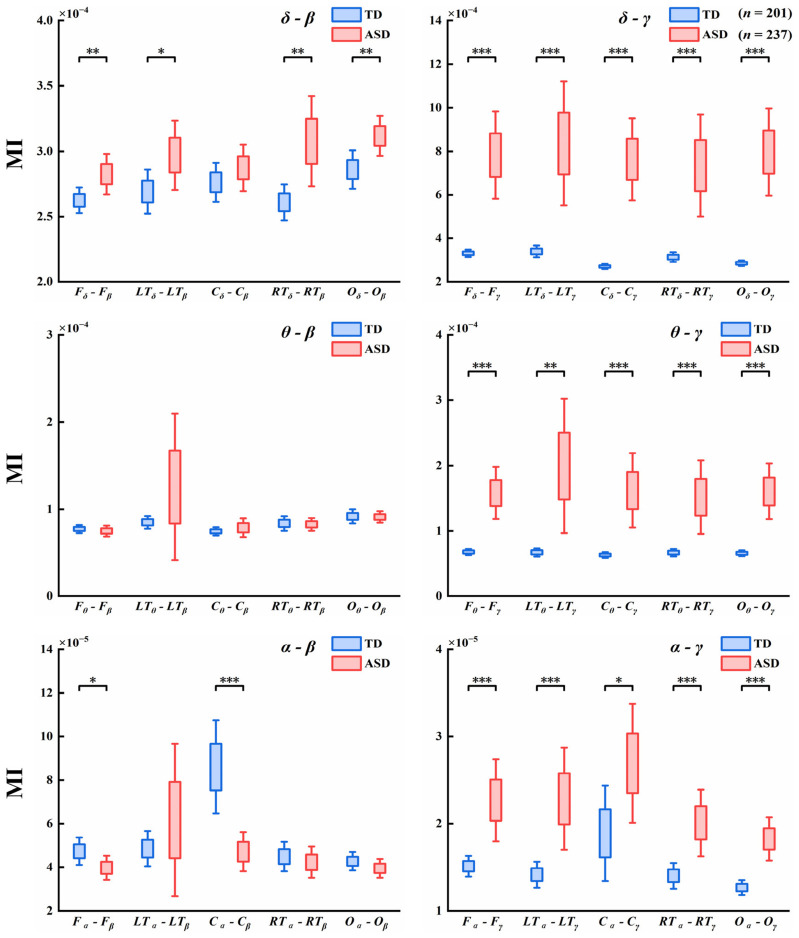
Box plots quantifying inter-group regional differences in intra-region PAC. The left panel illustrates the significant differences for the δ–β, θ–β, and α–β frequency couplings across brain regions. The right panel presents the differences for the δ–γ, θ–γ, and α–γ couplings in various regions. The top-left panel illustrates the δ–β PAC. The labels along the horizontal axis correspond to intra-region coupling relationships: *F_δ_-F_β_* represents the modulation of prefrontal β amplitude by the phase of prefrontal δ oscillations; *LT_δ_-LT_β_* indicates the modulation of left temporal β amplitude by left temporal δ phase; *C_δ_-C_β_* denotes the modulation of central β amplitude by central δ phase; *RT_δ_-RT_β_* reflects the modulation of right temporal β amplitude by right temporal δ phase; and *O_δ_-O_β_* signifies the modulation of occipital β amplitude by occipital δ phase. The horizontal axes of the other five panels follow the same labeling convention, but correspond to different frequency-band combinations for PAC analysis. All reported *p*-values were corrected for multiple comparisons using the Bonferroni method. Asterisks denote the level of statistical significance: * 0.01 < *p* < 0.05, ** 0.001 < *p* < 0.01, *** *p* < 0.001. Regions without annotations indicate no statistically significant difference (*p* > 0.05). Significant differences between the two groups were observed across multiple brain regions and frequency pairs, specifically including: *F_δ_-F_β_* (*p* = 0.005), *LT_δ_-LT_β_* (*p* = 0.023), *RT_δ_-RT_β_* (*p* = 0.001), *O_δ_-O_β_* (*p* = 0.002), *F_δ_-F_γ_* (*p* < 0.001), *LT_δ_-LT_γ_* (*p* < 0.001), *C_δ_-C_γ_* (*p* < 0.001), *RT_δ_-RT_γ_* (*p* < 0.001), *O_δ_-O_γ_* (*p* < 0.001), *F_θ_-F_γ_* (*p* < 0.001), *LT_θ_-LT_γ_* (*p* = 0.001), *C_θ_-C_γ_* (*p* < 0.001), *RT_θ_-RT_γ_* (*p* < 0.001), *O_θ_-O_γ_* (*p* < 0.001), *F_α_-F_β_* (*p* = 0.034), *C_α_-C_β_* (*p* < 0.001), *F_α_-F_γ_* (*p* < 0.001), *LT_α_-LT_γ_* (*p* < 0.001), *C_α_-C_γ_* (*p* = 0.030), *RT_α_-RT_γ_* (*p* < 0.001) and *O_α_-O_γ_* (*p* < 0.001).

By calculating and comparing the intra-region PAC strength between the TD group (Figure 2a) and the ASD group (Figure 2b) across all regions of interest, we identified significant frequency-dependent and region-specific differences (Figure 2c). Furthermore, we quantified the statistical significance of these differences for each brain region and frequency pair combination (Figure 3).

The results, presented in Figure 3, revealed a multi-regional and significant enhancement of PAC involving high-frequency γ-band amplitude in the ASD group compared to the TD group. Specifically, within the frontal, temporal, central, and occipital regions, the strength of δ–γ, θ–γ, and α–γ PAC was significantly higher in children with ASD than in TD controls (*p* < 0.05). This pattern of enhancement suggests that γ-band oscillations in the ASD cohort are subject to excessive modulation by low-frequency rhythms (δ, θ, α), an abnormality that is not confined to a single localized area but rather is observable across all sampled regions. For δ–β coupling, the ASD group also showed a significant increase in PAC strength. However, unlike the cross-regional consistency observed for the γ band, the enhancement of δ–β coupling displayed distinct spatial selectivity. The δ–β PAC was significantly higher in the ASD group within the frontal, temporal, and occipital lobes compared to the TD group. In contrast, no statistically significant between-group difference was found in the central region.

In contrast to the enhancement patterns described above, α–β PAC exhibited a significant reduction in the ASD group, which was highly regionally confined. Compared to the TD group, children with ASD showed significantly lower α–β PAC strength specifically in the frontal and central regions (*p* < 0.05). No significant between-group differences were detected in the temporal or occipital lobes. This finding suggests an impairment in the physiological regulatory mechanism whereby α oscillations modulate β-band activity within frontal and central regions in ASD. Notably, for θ–β coupling, no statistically significant differences were found between the ASD and TD groups in any of the examined regions—frontal, temporal, central, or occipital (*p* > 0.05). Detailed statistical results, including Bonferroni-corrected *p*-values and effect sizes (Cohen’s d) for each brain region and frequency pair, are summarized in Table 2.

### 3.3. Frequency-Specific Alterations in Inter-Region PAC

**Figure 4:**
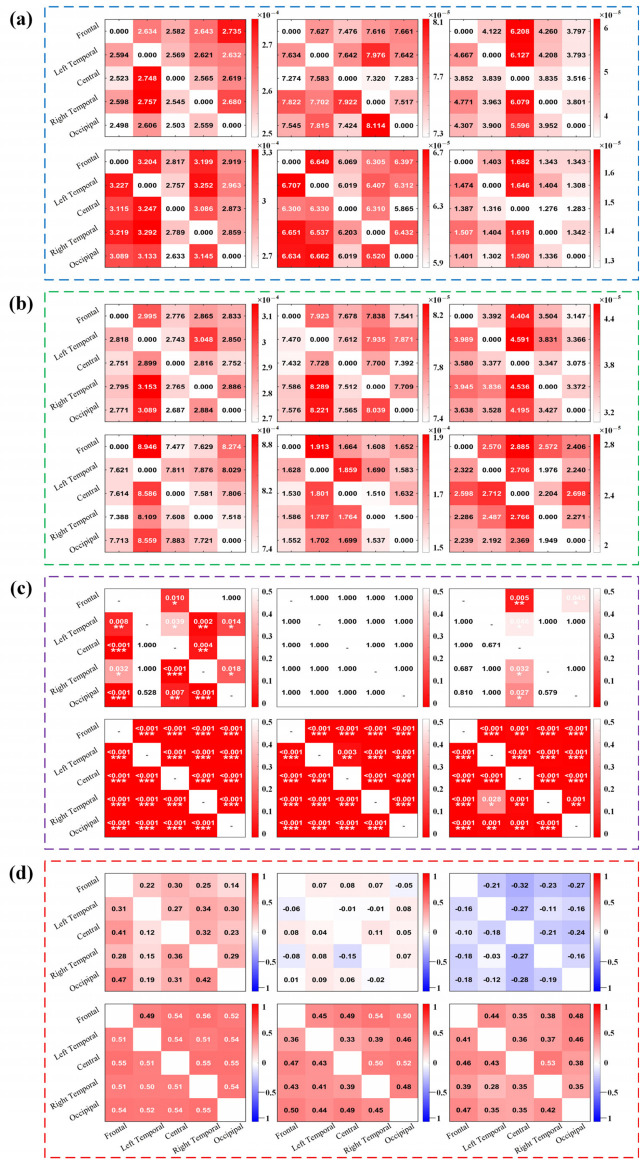
Heatmaps of inter-regional PAC strength, statistical significance, and effect sizes. Panel (**a**) illustrates the average inter-regional PAC strength matrices for the TD group, and panel (**b**) presents the corresponding matrices for the ASD group. In these matrices, the x-axis (columns) represents the source region providing the low-frequency phase, and the y-axis (rows) represents the target region providing the high-frequency amplitude. The top row of matrices corresponds to δ–β, θ–β, and α–β coupling, while the bottom row corresponds to δ–γ, θ–γ, and α–γ coupling. Panel (**c**) displays the between-group statistical significance matrices (ASD vs. TD, Bonferroni-corrected). Red cells indicate statistically significant differences; asterisks denote significance levels: * *p* < 0.05, ** *p* < 0.01, *** *p* < 0.001. Blank cells indicate no significant difference (*p* > 0.05). Panel (**d**) presents the effect size (Cohen’s d) matrices for each frequency pair. Warm colors (red) denote positive effect sizes, indicating enhanced PAC strength in the ASD group relative to the TD group, while cool colors (blue) denote negative effect sizes, indicating reduced PAC strength in the ASD group.

To investigate the specificity of inter-regional synchronization at the scalp level in the brains of children with ASD, we compared the inter-region PAC strength between the ASD and TD groups. Figure 4a,b presents the inter-region PAC strength matrices for the TD and ASD groups, respectively, across different frequency pairs. Figure 4c displays the corresponding matrix of between-group statistical significance after Bonferroni correction. Furthermore, Figure 4d illustrates the effect size (Cohen’s d) matrices, quantifying the magnitude of the differences between the ASD and TD groups.

The results are presented in Figure 4c. The inter-region PAC in the ASD group exhibited a pattern characterized by multi-regional increases in synchronization coexisting with weakened connectivity along specific pathways. Firstly, regarding coupling involving high-frequency γ-band amplitude, the ASD group demonstrated enhanced synchronization across the sampled regions. Specifically, for the δ–γ, θ–γ, and α–γ frequency pairs, the PAC strength for nearly all examined region pairs (e.g., frontal-occipital, temporal-central) was significantly higher in the ASD group than in the TD group (*p* < 0.05). Secondly, regarding δ–β coupling, while PAC was significantly enhanced in the ASD group across a majority of the analyzed inter-regional connections (e.g., frontal-temporal, central-temporal), no significant between-group differences were observed for connections linking the left temporal lobe to the remaining sampled sites, or along specific pathways such as right temporal–frontal, occipital–frontal, and occipital–central connections. Thirdly, α–β coupling demonstrated a highly selective decrease in connectivity. A significant reduction in α–β PAC was observed in the ASD group only for connections involving the central region with other brain areas and along the occipital–frontal pathway (*p* < 0.05). This spatial pattern aligns with the weakened α–β PAC found in the frontal and central regions in the intra-region analysis. Lastly, among all frequency pairs, θ–β coupling showed no significant between-group differences at the inter-region level.

### 3.4. Correlation Between Intra-Region PAC Abnormalities and Clinical Symptoms

**Figure 5:**
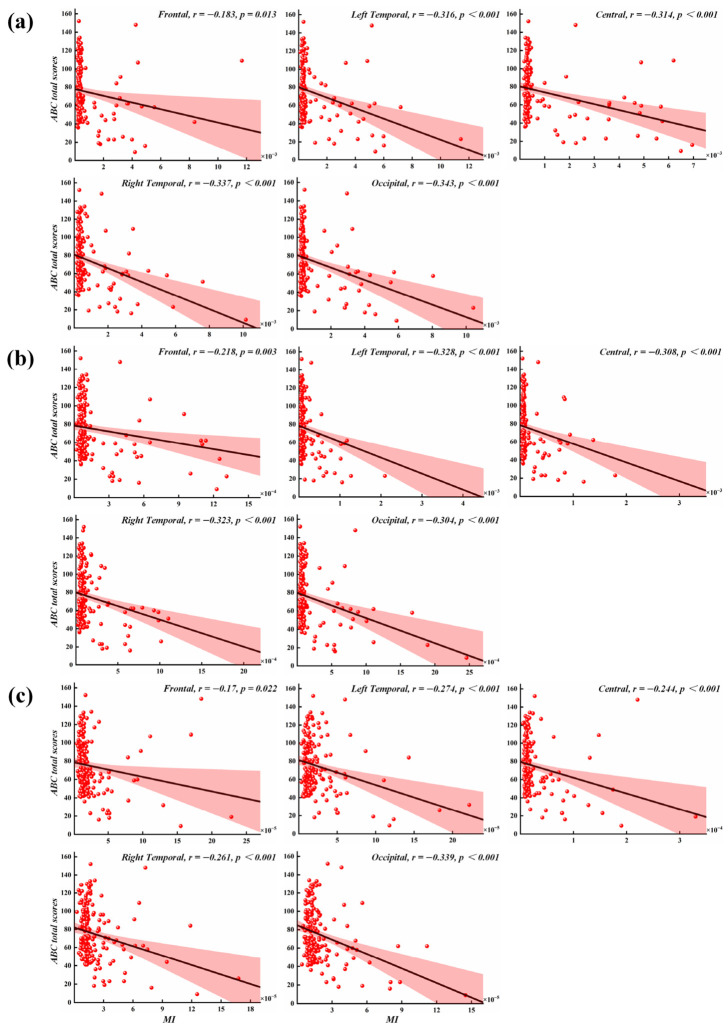
Correlation analysis between intra-region low-frequency–γ PAC strength and clinical behavioral scores. Scatter plots illustrate the linear relationships (Pearson correlation analysis) between the MI and the total ABC score: (**a**) displays the results of Pearson correlation analysis between δ–γ PAC strength and ABC scores across five brain regions: the frontal, left temporal, central, right temporal and occipital (arranged from left to right and top to bottom). (**b**) and (**c**) show the corresponding Pearson correlation analyses for θ–γ PAC and α–γ PAC with ABC scores, respectively, following the same spatial layout as in (**a**). Each red dot represents one participant with ASD. The solid black line indicates the linear regression fit, and the pink shaded area represents the 95% confidence interval.

**Figure 6:**
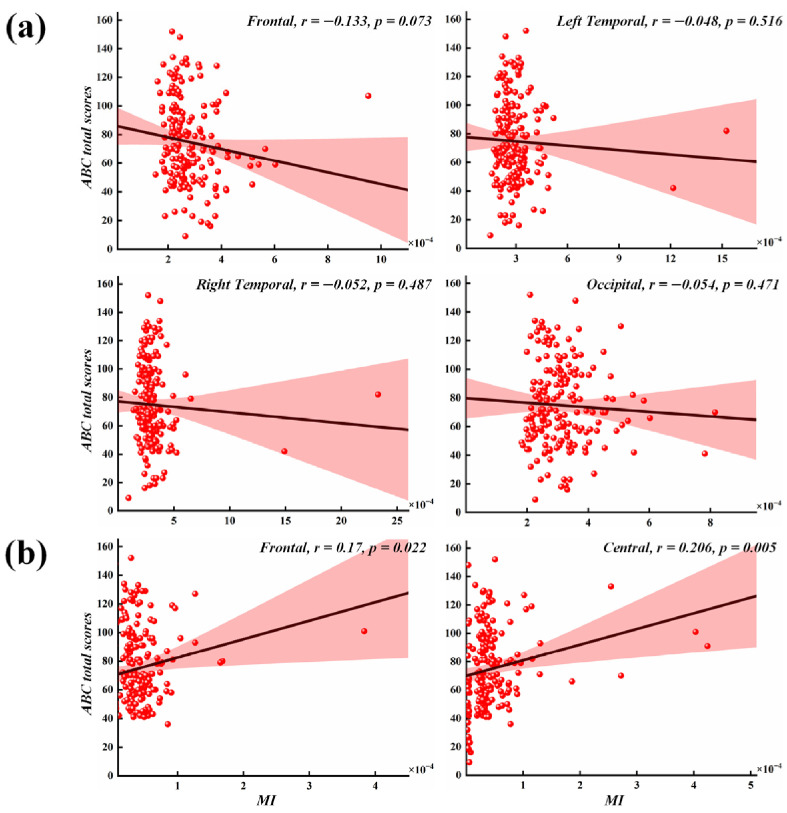
Correlation analysis between intra-region low-frequency–β PAC strength and clinical behavioral scores. Scatter plots illustrate the linear relationships (Pearson correlation analysis) between the MI and the total ABC score: (**a**) presents the results of Pearson correlation analysis between δ–β PAC strength and ABC scores across four brain regions: the frontal, left temporal, right temporal and occipital (arranged sequentially from left to right and top to bottom). (**b**) shows the corresponding analysis for α–β PAC strength versus ABC scores in two regions: the frontal and the central. Each red dot represents one participant with ASD. The solid black line indicates the linear regression fit, and the pink shaded area represents the 95% confidence interval.

To investigate the relationship between the observed electrophysiological abnormalities and clinical behavioral phenotypes, Pearson correlation analyses were conducted within the ASD group between PAC strength (for pairs showing significant between-group differences) and the total score on the ABC (Figure 5 and Figure 6, Table 3). The results revealed significant correlations between specific PAC metrics and the severity of behavioral symptoms, suggesting their potential value as biomarkers reflecting the clinical phenotype of ASD.

Figure 5a shows the correlation between δ–γ PAC strength and ABC scores. Significant negative correlations were found in the frontal (r = −0.183, *p* = 0.013), left temporal (r = −0.316, *p* < 0.001), central (r = −0.314, *p* < 0.001), right temporal (r = −0.337, *p* < 0.001), and occipital (r = −0.343, *p* < 0.001) lobes. Similarly, Figure 5b for θ–γ PAC revealed significant negative correlations across the same regions (frontal: r = −0.218, *p* = 0.003; left temporal: r = −0.328, *p* < 0.001; central: r = −0.308, *p* < 0.001; right temporal: r = −0.323, *p* < 0.001; occipital: r = −0.304, *p* < 0.001). Figure 5c for α–γ PAC also demonstrated significant negative correlations in all five regions (frontal: r = −0.171, *p* = 0.022; left temporal: r = −0.274, *p* < 0.001; central: r = −0.244, *p* < 0.001; right temporal: r = −0.261, *p* < 0.001; occipital: r = −0.339, *p* < 0.001).

Figure 6a presents the correlation for δ–β PAC. Although between-group comparisons indicated elevated δ–β PAC in multiple regions, its strength was not significantly correlated with ABC total scores (*p* > 0.05), suggesting no linear correspondence between δ–β hyper-coupling and the specific behavioral phenotypes assessed by the ABC scale. Figure 6b shows that α–β PAC strength was significantly positively correlated with ABC scores in the frontal (r = 0.170, *p* = 0.022) and central (r = 0.206, *p* = 0.005) regions.

Table 3 presents the Pearson correlation analysis between PAC strength and ABC scores. It is noteworthy that significant negative correlations were consistently observed for the δ–γ, θ–γ, and α–γ frequency pairs across all sampled scalp regions, suggesting that higher PAC strength in these bands is associated with lower ABC scores. Conversely, significant positive correlations were specifically found for the α–β pair in the frontal and central regions.

## 4. Discussion

This study systematically investigated resting-state PAC patterns in a large cohort of young children with ASD and TD controls using 8-channel EEG. Our findings reveal significant frequency-specific dysregulation of PAC in the ASD group, characterized by both hyper-coupling and regionally specific hypo-coupling patterns.

Our analysis revealed prominent hyper-coupling in the δ–β/γ, θ–γ, and α–γ frequency pairs across multiple brain regions (frontal, left temporal, central, right temporal, and occipital lobes). This enhanced temporal alignment indicates that the phase of slower oscillations excessively modulates the amplitude of faster oscillations in children with ASD compared to TD controls [9,13]. Conversely, we identified a regionally specific hypo-coupling pattern for α–β PAC, particularly in the frontal and central regions. Interestingly, our analysis detected no significant between-group differences in θ–β PAC across any evaluated region. These distinct patterns of dysregulation may suggest that neural oscillatory coordination in ASD does not alter uniformly but exhibits complex, frequency- and region-specific deviations from typical development at the scalp level [19].

Extending beyond intra-region dynamics, we evaluated inter-region PAC to assess synchronization across different scalp regions. Consistent with our intra-regional findings, our results demonstrated multi-regional and robust hyper-coupling in the δ–γ, θ–γ, and α–γ frequency pairs across nearly all inter-regional connections, alongside broadly enhanced δ–β coupling in the ASD group. This multi-regional hyper-coupling implies that the phase of slow oscillations in one cortical region excessively entrains the amplitude of fast oscillations in distant regions. Such an atypical pattern suggests a hyper-connected state of cross-frequency integration, which may hinder the brain’s ability to segregate distinct functional processes efficiently [5,20]. Conversely, we observed a distinct pattern of inter-regional hypo-coupling specifically within the α–β pair, primarily affecting connections involving the central region and the occipito-frontal pathway. This reduced inter-region coordination aligns with our intra-region findings and points to a deficit in inter-regional communication, likely affecting specific attentional or sensorimotor integration processes [12,21,22]. Furthermore, mirroring the intra-regional results, our analysis detected no significant inter-regional coupling differences in the θ–β band, reinforcing the frequency-specific nature of these topological alterations. These inter-region coordination differences suggest that ASD involves atypical oscillatory coupling that extends across disparate scalp regions, reflecting multi-regional surface electrophysiological anomalies [20,23].

Furthermore, we explored the linear relationships between PAC strength and clinical behavioral scores on the ABC. Our analysis revealed distinct, frequency-specific correlation patterns. Specifically, our results consistently demonstrated significant negative correlations between ABC total scores and the PAC strengths of δ–γ, θ–γ, and α–γ frequency pairs across all five regions of interest (frontal, left temporal, central, right temporal, and occipital). This seemingly paradoxical relationship, whereby a stronger hyper coupled state correlates with less severe clinical symptoms. Rather than viewing γ band hyper-coupling strictly as a disruptive pathological phenomenon, this negative correlation strongly suggests that it may represent an atypical but adaptive compensatory neural mechanism. Given the spatial constraints of our 8-channel EEG setup, this phenomenon is best understood through the lens of local cortical dynamics. Autism is widely associated with localized excitation/inhibition (E/I) imbalance and excessive high-frequency cortical noise [24]. In this context, the heightened modulation of γ amplitude by low-frequency phases might represent a rigid local temporal mechanism employed by the autistic brain to actively constrain and organize aberrant high-frequency bursts. Consequently, children who are capable of mounting this compensatory hyper synchronization to enforce strict local temporal coordination may better manage sensory and cognitive processing noise, thereby presenting with milder behavioral symptoms (lower ABC scores). This aligns with the perspective that not all electrophysiological deviations in ASD are inherently detrimental; some serve as active, adaptive coping strategies [25]. Conversely, a different pattern emerged for α–β PAC, which exhibited regionally specific positive correlations with ABC total scores exclusively within the frontal and central regions. This indicates that heightened α–β coupling in these anterior and sensorimotor areas may reflect greater behavioral symptom severity [26]. Additionally, although we observed alterations in δ–β PAC between groups, its strength did not show any statistically significant correlation with ABC total scores across any region, which may imply no direct linear correspondence between δ–β coupling and the specific behavioral phenotypes the ABC scale measures. However, we must note that the significant linear correlation coefficients remained relatively modest. This highlights the clinical heterogeneity of ASD and suggests that while scalp-level PAC metrics can capture certain aspects of neural coordination atypicalities, they explain only a portion of the complex behavioral variance.

## 5. Limitations

This study has several limitations. First, the 8-channel scalp EEG system used here has inherent limitations in spatial resolution. Although region-specific PAC abnormalities were identified, the precise localization of the deep neural sources underlying these aberrant coupling signals remains constrained by the volume conduction effects of scalp EEG and the limited number of recording channels. Future research should integrate high spatial resolution multimodal neuroimaging techniques, such as high-density EEG (e.g., 128- or 256-channel systems), MEG, and functional magnetic resonance imaging (fMRI). Employing these advanced methodologies is essential to validate our scalp-level observations at a finer spatial scale and to precisely delineate the specific neuroanatomical pathways and macroscopic cortical networks underlying the aberrant PAC patterns. Second, the ABC employed in this study relies on caregiver reports, which may be subject to limitations such as reporting bias, individual differences, and insufficient dimensional discriminability. Furthermore, our dataset lacks comprehensive multi-dimensional clinical profiling. While we applied stringent exclusion criteria regarding major neurological comorbidities (e.g., epilepsy, traumatic brain injury) and concurrent use of neuroactive medications, detailed metrics regarding intellectual functioning (IQ), specific comorbid psychiatric conditions (such as ADHD or anxiety disorders), and sleep quality were not systematically collected. These uncontrolled variables, including IQ, psychiatric comorbidities, and sleep quality, could confound the observed EEG rhythms. Since these uncontrolled variables could potentially confound the observed EEG rhythms. Future studies should integrate objective behavioral assessment tasks and comprehensive clinical assessments, including standardized cognitive testing and psychiatric comorbidity screening, to provide a more nuanced explanation of the relationship between electrophysiological indices and specific functional phenotypes, thereby isolating the exact specificity of these EEG biomarkers to the core pathophysiology of ASD. Furthermore, the relatively modest linear correlation coefficients observed between PAC strength and ABC scores in the present study indicate that PAC can only explain a portion of the behavioral variance. This suggests that ASD symptoms are likely regulated by multiple, overlapping neural mechanisms. Third, the participants in this study span a broad developmental window (3–9 years of age), a period characterized by profound maturation of neural oscillatory dynamics, particularly the development of the α rhythm and β synchronization. While we ensured strict group-level age matching between the ASD and TD cohorts, age matching alone is insufficient to entirely eliminate developmental confounding. The inherent age-related variance within this 6-year span could potentially interact with or obscure more nuanced, age-specific PAC anomalies. In future studies, we aim to stratify the cohort into narrower developmental stages—such as preschool (3–6 years) and school-age (6–9 years)—or even conduct year-by-year analyses, to accurately map the precise neurodevelopmental trajectories of PAC in early childhood. Fourth, although our preprocessing pipeline (EEMD-ICA) and analytical metric (KL divergence) were explicitly chosen to minimize artifacts, we did not employ a formal surrogate-data validation procedure (e.g., time-shifting surrogates). Therefore, we cannot mathematically rule out all potential spurious coupling generated by the geometric asymmetry of non-sinusoidal low-frequency oscillations. Future investigations with smaller cohorts or higher computational resources should incorporate strict surrogate statistical testing to further validate the genuineness of these cross-frequency neural interactions. Finally, this study employed a cross-sectional design, which precludes direct insights into the dynamic trajectory of the observed PAC abnormalities throughout the neurodevelopment of ASD. Therefore, we cannot determine whether these aberrant coupling features represent a risk marker for the onset of ASD, a developmental characteristic, or an outcome of long-term adaptive changes. Future longitudinal follow-up studies, focusing on narrower age bands and tracking the evolution of PAC patterns in individuals at different developmental stages, would be invaluable for clarifying its temporal characteristics and potential clinical value within the neurodevelopmental course of ASD, and for better understanding age-related influences.

## 6. Conclusions

This large-sample study demonstrates that young children with ASD exhibit distinct, frequency-specific alterations in PAC strength at the scalp level. While our low-density EEG setup constrains the inference of macroscopic networks, it robustly captures vital intra-region and inter-region temporal coordination anomalies. Notably, intra-region PAC strength is significantly correlated with ABC scores. Specifically, increased low-frequency to γ-band modulation was negatively correlated with symptom severity, suggesting that this hyper-coupling may serve as an atypical but adaptive local compensatory mechanism rather than a mere pathological deficit. Conversely, reduced α–β PAC strength in the prefrontal and central regions was positively associated with higher ABC scores. Together, these scalp-level electrophysiological markers provide a nuanced perspective on the complex, adaptive neural dynamics underlying early ASD development.

## Figures and Tables

**Table 1:** Demographic Characteristics of the Study Participants.

Characteristics	TD Group (*n* = 201)	ASD Group (*n* = 237)
**Gender (*n*)**		
Male	154	183
Female	47	54
**Age (years)**		
Mean (SD)	5.26 (1.23)	5.33 (1.41)
Range (Min–Max)	3.12–8.87	3.13–8.95

**Table 2:** Statistical Differences in PAC Strength Between Children with ASD and TD Controls Across Brain Regions.

Coupling Pair	Scalp Region	*p* Value	*Cohen’s d*
**delta–beta**	Frontal	0.005	0.209
	Left Temporal	0.023	0.246
	Central	0.776	0.087
	Right Temporal	0.001	0.354
	Occipital	0.002	0.220
**delta–gamma**	Frontal	<0.001	0.464
	Left Temporal	<0.001	0.509
	Central	<0.001	0.541
	Right Temporal	<0.001	0.525
	Occipital	<0.001	0.530
**theta–beta**	Frontal	0.863	−0.056
	Left Temporal	0.748	0.141
	Central	0.852	0.070
	Right Temporal	0.870	−0.025
	Occipital	0.846	−0.012
**theta–gamma**	Frontal	<0.001	0.466
	Left Temporal	0.001	0.378
	Central	<0.001	0.460
	Right Temporal	<0.001	0.441
	Occipital	<0.001	0.459
**alpha–beta**	Frontal	0.034	−0.161
	Left Temporal	0.223	0.106
	Central	<0.001	−0.281
	Right Temporal	0.436	−0.068
	Occipital	0.543	−0.099
**alpha–gamma**	Frontal	<0.001	0.317
	Left Temporal	<0.001	0.415
	Central	0.030	0.171
	Right Temporal	<0.001	0.419
	Occipital	<0.001	0.431

The statistical results presented were derived from independent-samples *t*-tests, with *p*-values corrected using the Bonferroni procedure. The analysis revealed a pattern of widespread hyper-coupling in the δ–β/γ, θ–γ, and α–γ frequency pairs. In contrast, a regionally specific hypo-coupling pattern was observed for α–β coupling in the frontal and central regions.

**Table 3:** Correlation Analysis Between PAC Strength and ABC Scores in Children with ASD.

PAC Pair	Frontal	Left Temporal	Central	Right Temporal	Occipital
***δ-β***	−0.133	−0.048	—	−0.052	−0.054
***δ-γ***	−0.183 *	−0.316 ***	−0.314 ***	−0.337 ***	−0.343 ***
***θ-γ***	−0.218 **	−0.328 ***	−0.308 ***	−0.323 ***	−0.304 ***
***α-β***	0.170 *	—	0.206 **	—	—
***α-γ***	−0.171 *	−0.274 ***	−0.244 ***	−0.261 ***	−0.339 ***

Note. Values represent Pearson correlation coefficients (*r*). Asterisks indicate statistical significance (* *p* < 0.05, ** *p* < 0.01, *** *p* < 0.001, Bonferroni corrected). A dash (—) denotes regions not included in the analysis.

## Data Availability

The data that supports the findings of this study are available from the corresponding author upon reasonable request.

## References

[1] First M.B., Yousif L.H., Clarke D.E., Wang P.S., Gogtay N., Appelbaum P.S. (2022). DSM-5-TR: Overview of what’s new and what’s changed. World Psychiatry.

[2] Lord C., Brugha T.S., Charman T., Cusack J., Dumas G., Frazier T., Jones E.J.H., Jones R.M., Pickles A., State M.W. (2020). Autism spectrum disorder. Nat. Rev. Dis. Primers.

[3] McPartland J.C., Bernier R.A., Jeste S.S., Dawson G., Nelson C.A., Chawarska K., Earl R., Faja S., Johnson S.P., Sikich L. (2020). The autism biomarkers consortium for clinical trials (ABC-CT): Scientific context, study design, and progress toward biomarker qualification. Front. Integr. Neurosci..

[4] Garcés P., Baumeister S., Mason L., Chatham C.H., Holiga S., Dukart J., Jones E.J.H., Banaschewski T., Baron-Cohen S., Bölte S. (2022). Resting state EEG power spectrum and functional connectivity in autism: A cross-sectional analysis. Mol. Autism.

[5] Hahamy A., Behrmann M., Malach R. (2015). The idiosyncratic brain: Distortion of spontaneous connectivity patterns in autism spectrum disorder. Nat. Neurosci..

[6] Canolty R.T., Knight R.T. (2010). The functional role of cross-frequency coupling. Trends Cogn. Sci..

[7] Tort A.B., Komorowski R., Eichenbaum H., Kopell N. (2010). Measuring phase-amplitude coupling between neuronal oscillations of different frequencies. J. Neurophysiol..

[8] Berman J.I., Liu S., Bloy L., Blaskey L., Roberts T.P., Edgar J.C. (2015). Alpha-to-gamma phase-amplitude coupling methods and application to autism spectrum disorder. Brain Connect..

[9] Peck F., Naples A.J., Webb S.J., Bernier R.A., Chawarska K., Dawson G., Faja S., Jeste S., Murias M., Nelson C.A. (2022). Phase-Amplitude Coupling in Autism Spectrum Disorder: Results from the Autism Biomarkers Consortium for Clinical Trials. MedRxiv.

[10] Kroupi E., Jones E.J., Oakley B., Buitelaar J., Charman T., Loth E., Murphy D., Soria-Frisch A. (2024). Age-related differences in delta-beta phase-amplitude coupling in autistic individuals. Clin. Neurophysiol..

[11] Seymour R.A., Rippon G., Gooding-Williams G., Schoffelen J.M., Kessler K. (2019). Dysregulated oscillatory connectivity in the visual system in autism spectrum disorder. Brain.

[12] Khan S., Michmizos K., Tommerdahl M., Ganesan S., Kitzbichler M.G., Zetino M., Garel K.-L.A., Herbert M.R., Hämäläinen M.S., Kenet T. (2015). Somatosensory cortex functional connectivity abnormalities in autism show opposite trends, depending on direction and spatial scale. Brain.

[13] Port R.G., Dipiero M.A., Ku M., Liu S., Blaskey L., Kuschner E.S., Edgar J.C., Roberts T.P., Berman J.I. (2019). Children with autism spectrum disorder demonstrate regionally specific altered resting-state phase–amplitude coupling. Brain Connect..

[14] Guha M. (2014). Diagnostic and Statistical Manual of Mental Disorders: DSM-5.

[15] Zeng K., Chen D., Ouyang G., Wang L., Liu X., Li X. (2015). An EEMD-ICA approach to enhancing artifact rejection for noisy multivariate neural data. IEEE Trans. Neural Syst. Rehabil. Eng..

[16] Ma L., Liu W., Hudson A.E. (2019). Propofol anesthesia increases long-range frontoparietal corticocortical interaction in the oculomotor circuit in macaque monkeys. Anesthesiology.

[17] Zhang R., Ren Y., Liu C., Xu N., Li X., Cong F., Ristaniemi T., Wang Y. (2017). Temporal-spatial characteristics of phase-amplitude coupling in electrocorticogram for human temporal lobe epilepsy. Clin. Neurophysiol..

[18] Liang Z., Jin X., Ren Y., Yu T., Li X. (2021). Propofol anesthesia decreased the efficiency of long-range cortical interaction in humans. IEEE Trans. Biomed. Eng..

[19] O’Reilly C., Lewis J.D., Elsabbagh M. (2017). Is functional brain connectivity atypical in autism? A systematic review of EEG and MEG studies. PLoS ONE.

[20] Voytek B., Knight R.T. (2015). Dynamic network communication as a unifying neural basis for cognition, development, aging, and disease. Biol. Psychiatry.

[21] Engel A.K., Fries P. (2010). Beta-band oscillations—Signalling the status quo?. Curr. Opin. Neurobiol..

[22] Keehn B., Müller R.A., Townsend J. (2013). Atypical attentional networks and the emergence of autism. Neurosci. Biobehav. Rev..

[23] Sadaghiani S., Brookes M.J., Baillet S. (2022). Connectomics of human electrophysiology. NeuroImage.

[24] Dickinson A., Jones M., Milne E. (2016). Measuring neural excitation and inhibition in autism: Different approaches, different findings and different interpretations. Brain Res..

[25] Livingston L.A., Colvert E., Bolton P., Happé F., Social Relationships Study Team (2019). Good social skills despite poor theory of mind: Exploring compensation in autism spectrum disorder. J. Child Psychol. Psychiatry.

[26] Uddin L.Q. (2021). Cognitive and behavioural flexibility: Neural mechanisms and clinical considerations. Nat. Rev. Neurosci..

